# A novel sustainable biocide against the fruit fly *Drosophila suzukii* made from orange peels

**DOI:** 10.1038/s41598-024-75365-6

**Published:** 2024-11-14

**Authors:** Giovanni Davide Barone, Manfred Hartbauer

**Affiliations:** Department of Biology, University of Graz, Universitätsplatz 2, 8010 Graz, Austria

**Keywords:** Eco-friendly, Fruit fly control, Upcycling, Salty extract, Attract-and-kill approach, Choice experiments, Ecology, Agroecology, Invasive species

## Abstract

*Drosophila suzukii* (*D. suzukii*), a pervasive pest originating from Southeast Asia, presents a substantial risk to global agriculture. The ability of the female flies to lay eggs within fruits of varying maturity stages, combined with the accelerated offspring development within warmer climates, results in rapid population growth. This poses significant challenges for fruit production and viticulture, exacerbated by the increasing prevalence of pesticide resistance. We propose a solution to this growing issue using an attract-and-kill approach by making use of upcycled organic waste materials made from orange peels. Specifically, we have tested an innovative salty orange peel product (OPP) in a choice experiment, in which OPP and hydrogel (control) were made available to fruit flies in Petri dishes situated beneath red wine grapes. The number of dead flies in both Petri dishes were counted each day and fly maggots inside berries were extracted after four days. Since Petri dishes were covered with a red lid, flies only selected on the basis of olfactory cues. Our results showed a higher number of captured flies in Petri dishes containing OPP compared to those with the hydrogel control. Furthermore, a notable reduction in the number of maggots was observed inside grapes located above OPP compared to the grapes closer to the hydrogel control. Dilution of OPP was followed by a lower count of dead flies. In additional choice experiments, the concentration of NaCl was found to be positively correlated with the number of dead flies. This suggests an important lethal effect caused by high salt concentrations. In a final experiment, OPP was also compared to the commercially-available attractant called Drosalure™, which resulted in a slightly higher attractiveness of OPP to *D. suzukii*. These findings suggest that OPP holds potential as a cost-efficient and eco-friendly biocide made from organic waste material. OPP offered in attract-and-kill traps equipped with small entry holes is safe for bees and may replace other less eco-friendly control measures for *D. suzukii* in organic vineyards.

## Introduction

On a global dimension, there exist about 1,500 *Drosophila* species commonly known as vinegar flies^1^. One of the most problematic species among them is *Drosophila suzukii* Matsumura (*D*. *suzukii*), a species that has been accidentally transported to many continents, where fly populations quickly grew in the absence of natural enemies. In Japan, *D. suzukii* is better known as cherry drosophila, and in North America this fruit fly species was named spotted wing drosophila^2–4^ because males display two characteristic black spots on their front wings. *Drosophila suzukii* has been observed on wild strawberries and cultivated cherries for the first time in mainland Japan in 1916 and later between 1930 and 1931 in China and Korea^5–7^ Even now, this fruit fly species is still considered as a pest for soft and stone fruits in Japan^8^ and China^9,10^. During the last decades, this fly species has also been spotted in Great Britain^11^, Poland^12^, Hungary^13^, Italy^14–16^, Austria^17,18^, South America^19^, Mexico^20^, and other countries^21^, with many negative ecological and economic consequences. More recently, *D*. *suzukii* has also been reported in sub-Saharan Africa^22,23^ and Algeria^24^. One reason for its fast reproduction is related to the oviposition behavior of females, mostly infesting red-colored fruits in almost all ripening stages^7,25^ with the help of a serrated ovipositor that causes physical damage to the fruit^4^. The females preferentially lay their eggs into healthy fruits with a spherical surface and small radius^26,27^. Once the fly maggots hatch, the fruit begins to rot, which leads to reduced crop yield and significant financial losses^4,28–30^. As a consequence of this oviposition behavior, injury of the fruit often provides access to microbial infections causing further fruit damage^31–33^.

Certain chemical insecticides (e.g., azadirachtin®, chlorpyrifos®, cyazypyr®, dimethoate, Imidacloprid, lambda-cyhalothrin, Spinosad, and malathion) have proven to be effective against *D. suzukii*^34–36^. However, all these pesticides can have negative consequences for a large group of non-target insect species, some of them known as important pollinators^4^. In recent years, the contact and ingestion toxin Spinosad was extensively used for the control of *D. suzukii* in different agricultures (e.g.,^37^). This toxin is made from the bacterial species *Saccharopolyspora spinosa* and can also be used in organic farming, but no longer than three to four weeks before harvest. A recent study reported that overuse can be problematic because Spinosad resistance is emerging in a *D. suzukii* population in California^38^ and additional insecticide resistance management strategies are urgently needed to combat this pest in future. Another sustainable control method is based on spraying Silicium-oxide (diatomaceous earth) together with a wetting agent to reduce *D. suzukii* infestations^37^. However, this treatment turns orchards and wine yards into strange agricultural areas with a white color, but more problematic, the next rainfall requires another spray treatment.

In recent years, trends towards sustainable pest control strategies have strongly increased the attention of researchers (e.g.,^37,39–41^). Despite of known natural substances that are effective against *D. suzukii* (e.g.,^42–44^), only few registered basic substances are available as lure (e.g., ethanol and acetic acid) and insecticide against *D*. *suzukii* in Europe and, therefore, more sustainable control measures are urgently needed. One important aspect in viticulture is that certain plant species growing in the near of wine yards (e.g., in forests) favor *D. suzukii* invasions at certain times of the season^45,46^. Another study revealed that certain plant species can be used for trap cropping because this fly species prefers their fruits over the grapes of red wine^47^. In this study, we tested a biocidal solution for the control of *D. suzukii* in viticulture that is based on the upcycling of organic industrial waste material because large quantities of effective substances are needed for fruit fly control in vineyards.

In eco-friendly agriculture, it is mandatory to use control methods with high selectivity for the target organism in order to create a low impact on the ecosystem. Furthermore, control methods should be harmless for humans as well as for the host plants suffering from problematic organisms. Instead of spraying host plants (pesticide-based control), we suggest an attract-and-kill approach that can be realized with the help of fly traps that are equipped with small holes that restrict access to the lure. This biocidal approach is safe for bees and other insects with body sizes larger than fruit flies. In our laboratory study, we tested a novel lure for this kind of attract-and-kill approach which is upcycled from orange peels. This orange peel product (OPP) was created by submersing orange peel snippets into a salty solution before application of mechanical pressure that extracts orange peel oil. Fontana^48^ reported that essential oils of orange peels from Italy contain high amounts of D-limonene (> 95%), a natural mono-terpene that is known to be highly attractive and toxic for the Mediterranean fruit fly species *Bactrocera dorsalis*^49,50^. Additionally, orange peel oil contains about 1%—3% β-myrcene and 0.4%—5.6% β-linalool, both of these mono-terpenes are known to be attractive to *D. suzukii*^51,52^. These terpenes have an insecticidal effect against stored-product beetles^53,54^. Additionally, orange peels also contain small quantities of neral (< = 1%), a terpenoid with insecticidal activity against three mosquito species^55^. All these insecticidal substances may prevent fruit flies from exiting traps, once getting into contact with OPE.

To test the attractiveness and toxicity of OPP for *D. suzukii*, we performed choice experiments with ripe red wine grapes inside a closed terrarium. In this experiment, 40 fly individuals were offered the choice between two ripe red wine grapes that were fixed above a Petri dish containing either OPP or hydrogel that was used as control. We performed additional choice tests in a similar setup to study fly count after dilution of OPP or after reducing the salt concentration used for the submersion of the pieces of orange peels. Finally, we compared OPP in a choice setup with the commercially-available lure called Drosalure™ containing red wine, vinegar and yeast.

## Results

**Fig. 1:**
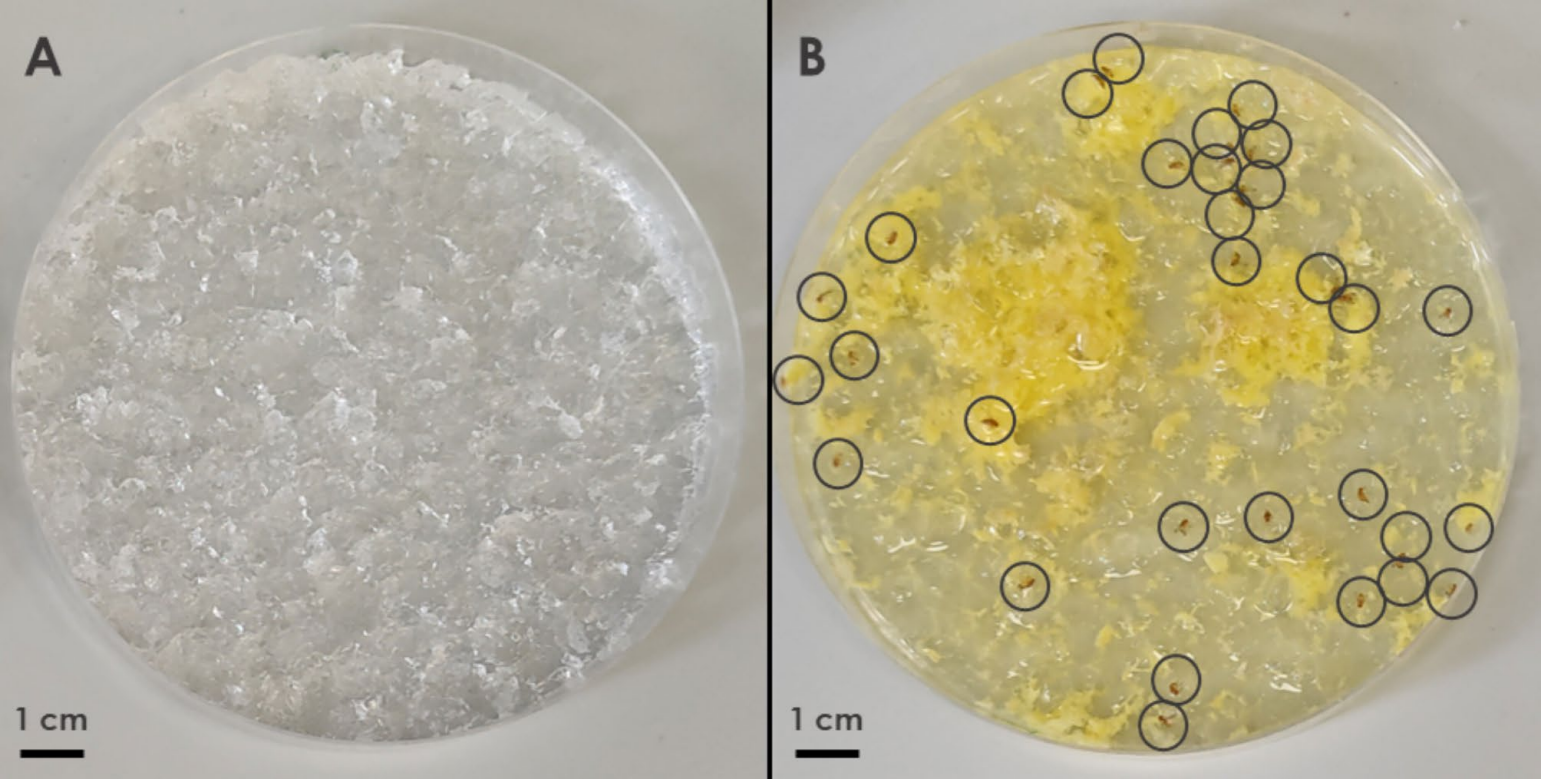
Representative example for the attractiveness of OPP in choice tests. Pictures of Petri dishes containing either only hydrogel (**A**) or OPP added on top of the hydrogel (**B**) after 4 days of exposure to fruit flies in a terrarium where also two red wine grapes were present. In this example, OPP was prepared 3 days before the start of the experiment and 40 D. suzukii individuals were released in the middle of the terrarium at the start of this experiment.

**Fig. 2:**
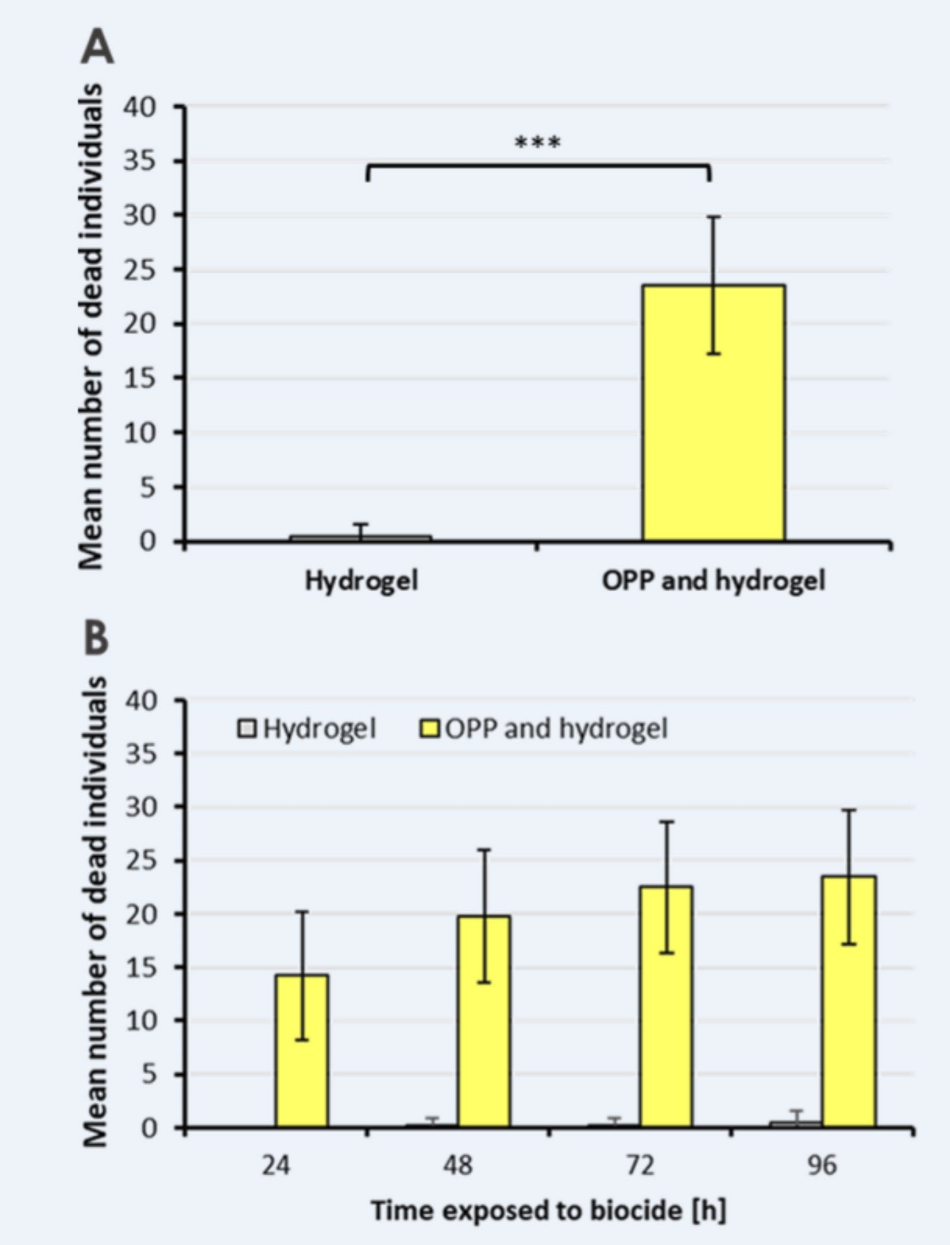
Mean number of dead fly individuals found inside Petri dishes in choice tests. (**A**) Mean number of dead fly individuals counted after 4 days in each of the two Petri dishes (paired t-test, p < 0.0001, N = 10). (**B **) Mean number of dead flies counted on each day (N = 10). 40 D. suzukii individuals were released at the beginning of each replicate in the middle of the terrarium. OPP was prepared 3 days before to the start of the experiment.

**Fig. 3:**
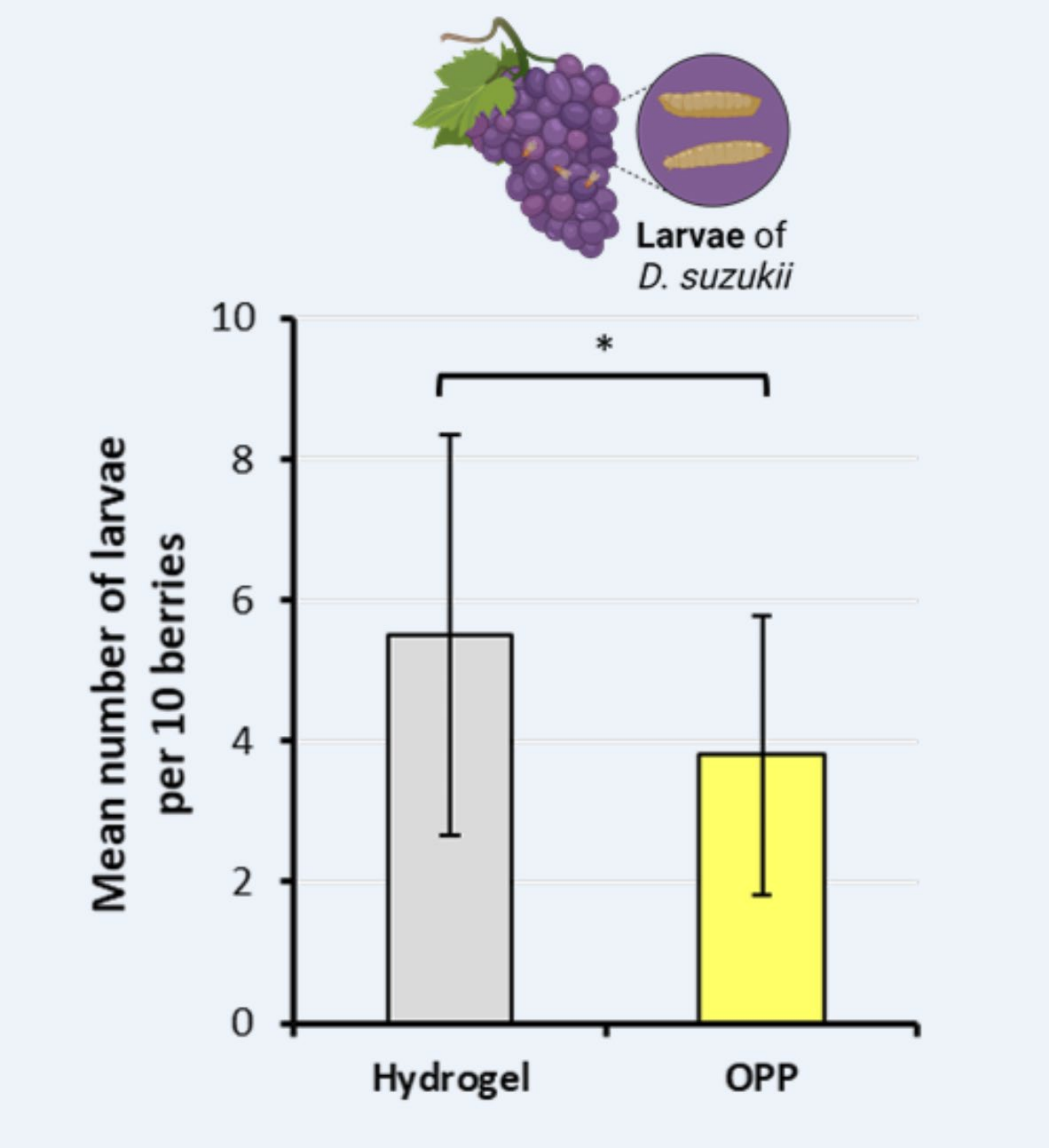
Mean number of larvae counted in bunches of grapes positioned above the Petri dishes. Two bunches of grapes consisting of 10 berries were positioned above the Petri dishes containing either only hydrogel or hydrogel with OPP. The number of fly larvae were counted after 4 days after the start of the choice experiment. Note that the berries located above the OPP contained a significantly lower number of larvae (paired t-test, p < 0.05, N = 10). 40 D. suzukii individuals were released at the beginning of each experiment in the middle of the terrarium.

Following a four-day period, it was evident that the number of deceased *D. suzukii* flies was significantly higher on the OPP side compared to the hydrogel control side, as illustrated in Fig. 1. In a total of 10 choice experiments, we consistently observed a significantly higher average number of deceased flies in the OPP Petri dishes compared to the hydrogel control side after four days (paired t-test, p < 0.0001, N = 10; Fig. 2A). The application of OPP in choice experiments led to a significant reduction in the larvae count in the berries positioned above the OPP in comparison to the berries closer to the hydrogel control (paired t-test, p < 0.05, N = 10; Fig. 3).

To analyze the time-dependent impact of the OPP insecticide, we recorded the daily count of deceased fruit flies throughout the experiment. The average number of dead fruit flies in both Petri dishes for each day is shown in Fig. 2B. Approximately 50% of *D. suzukii* flies died within the initial 48 h following exposure to OPP (Fig. 2B, N = 10). Remarkably, there was no significant increase in the number of deceased flies beyond three days from the beginning of the experiment.

**Fig. 4:**
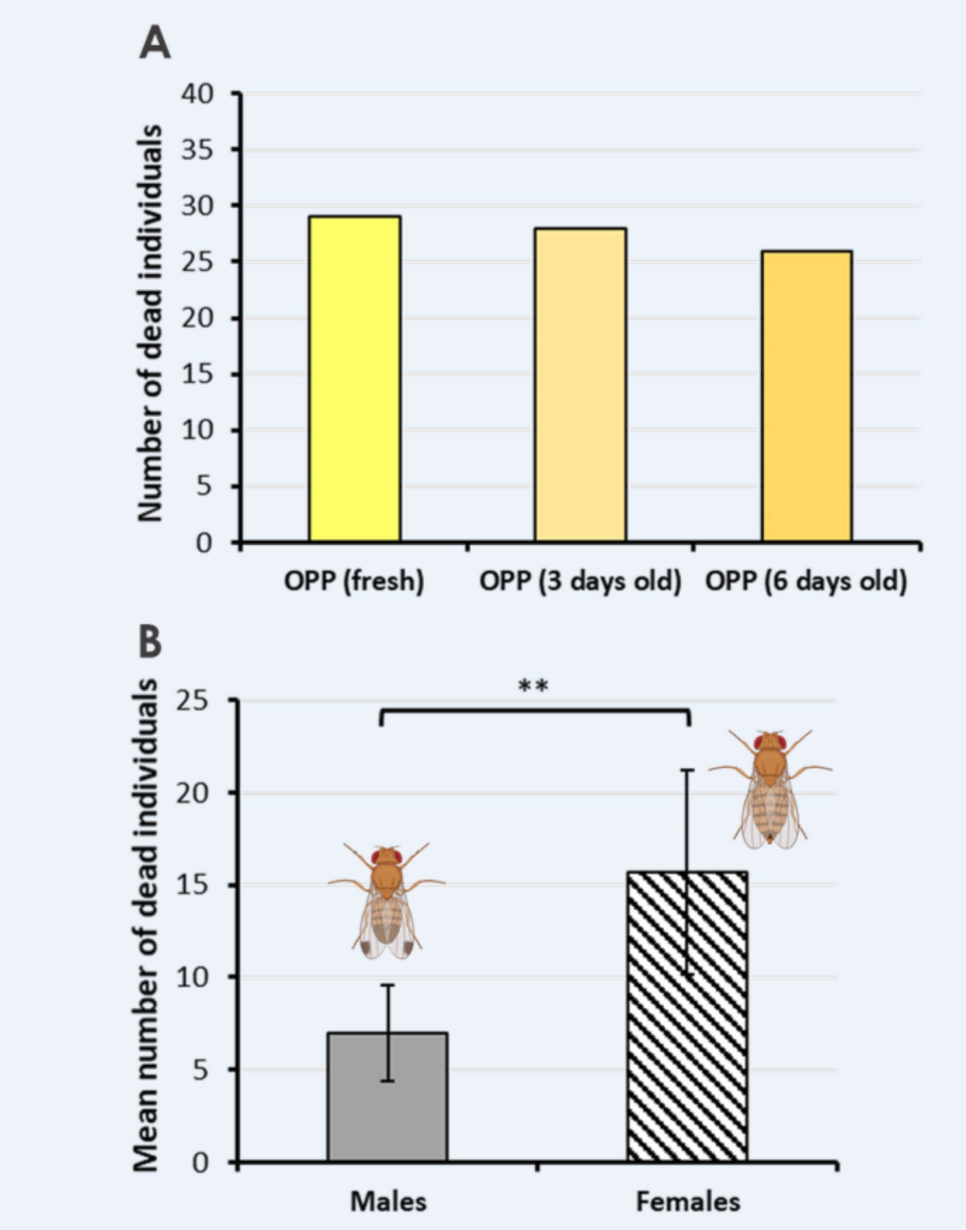
Comparison of the effectiveness of fresh and older OPP, and the sex of dead fly individuals. (**A**) Number of dead D. suzukii individuals found on fresh and several days old OPP extract. The oldest extract was prepared 6 days before the start of the experiment and the number of dead flies was counted 4 days after the start of this experiment. (**B**) The mean number of dead male (light gray bar) and female fly individuals (stripped bar) found dead on the OPP hydrogel after 4 days (paired t-test, p < 0.01, N = 7). 20 adult D. suzukii individuals of each sex were released at the beginning of each experiment in the middle of the terrarium.

In additional choice experiments, we examined the efficiency of freshly prepared OPP in comparison to OPP that had been prepared several days earlier. As shown in Fig. 4A, we observed a slight decrease in the number of deceased flies when using OPP prepared 6 days before the start of the choice experiment, in contrast to freshly prepared OPP. Interestingly, we noted a significantly higher count of deceased female flies on the OPP hydrogel in comparison to deceased males (paired t-test, p < 0.01, N = 7; Fig. 4B), which indicates a greater attraction of female flies to OPP, given that an equal number of males and females were released at the beginning of the choice experiments.

**Fig. 5:**
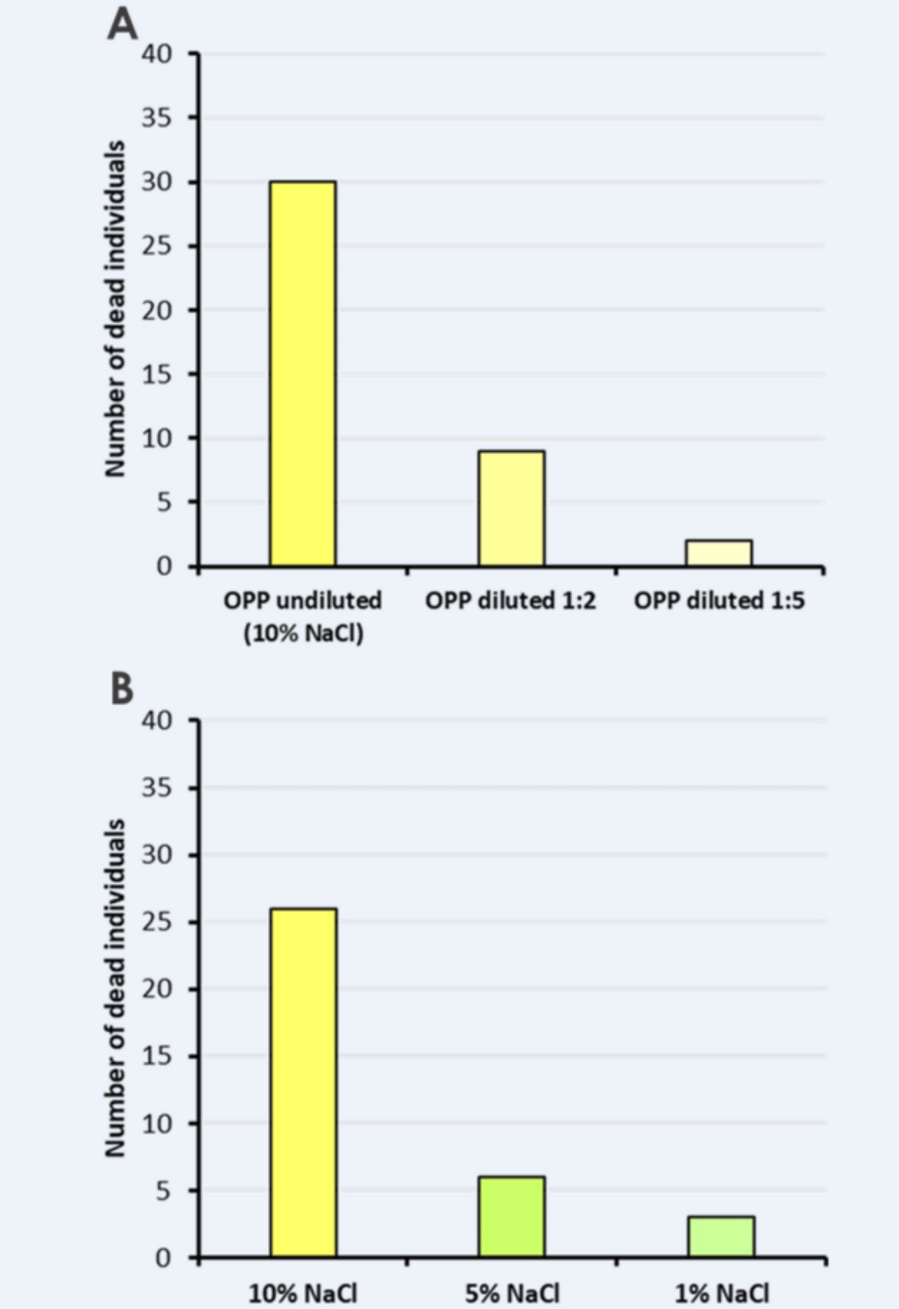
Effect of OPP dilution and reduced NaCl concentration on the number of dead flies. (**A**) The number of dead D. suzukii individuals counted on top of the OPP hydrogel after 4 days, when it was either offered fresh or 1:2 and 1:5 diluted with tap water. (**B**) The number of dead D. suzukii individuals counted after 4 days on top of the OPP hydrogel when different concentrations of NaCl were used for the extraction of OPP from orange peels.

In choice experiments conducted with 1:2 and 1:5 diluted OPP, a substantial decrease in the number of deceased flies was observed four days after the start of the experiment in comparison to undiluted OPP (Fig. 5A). Similarly, a decline in the number of deceased flies was noted following a reduction in the salt (NaCl) concentration in the solution used to submerge orange peel snippets (Fig. 5B). In this ‘salt experiment’, the highest number of *D. suzukii* flies was observed when orange peels were submerged in a 10% NaCl solution, while the lowest count was recorded in the experiment conducted with a 1% NaCl solution.

**Fig. 6:**
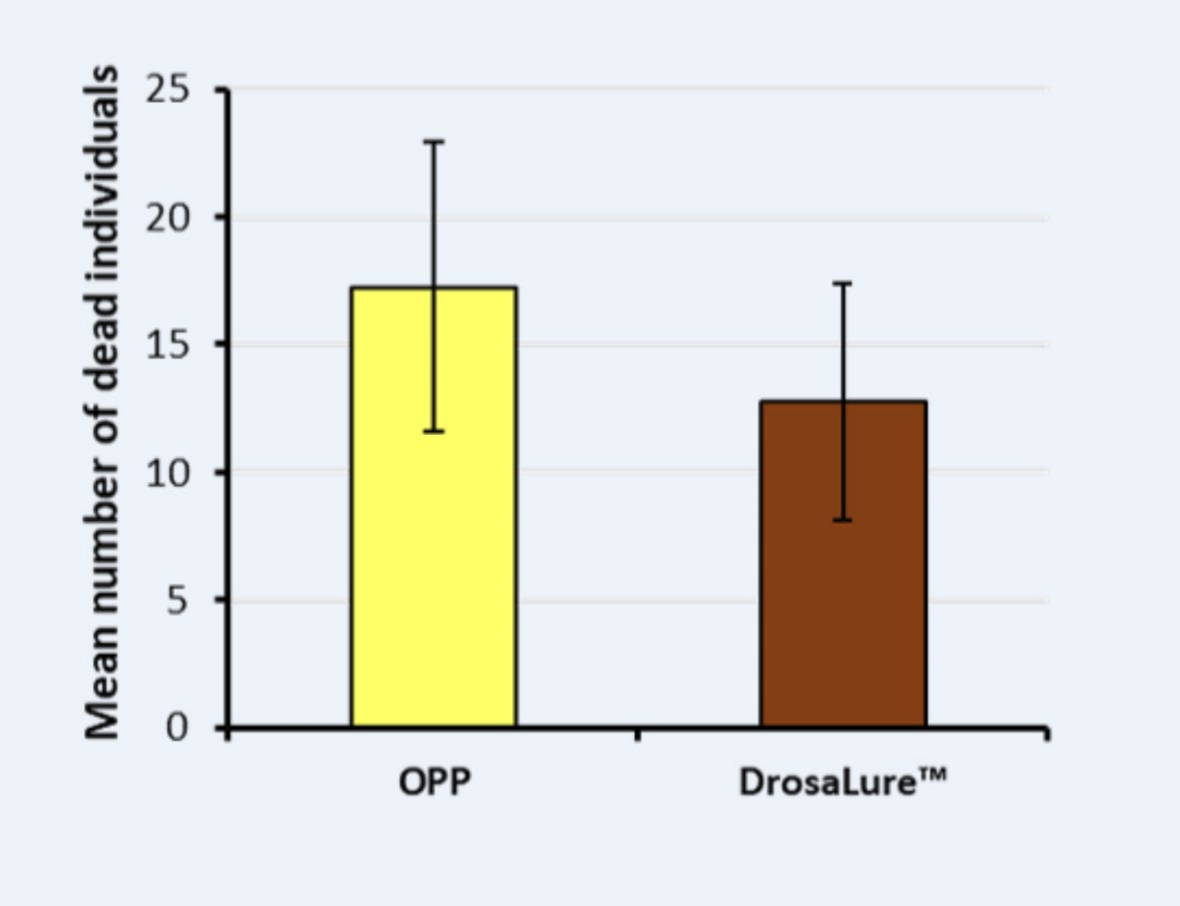
Comparison of the attractiveness of DrosalureTM with OPP in a choice experiment. Two bunches of grapes consisting of 10 berries were positioned above the Petri dishes containing hydrogel with OPP or liquid DrosalureTM. There was no significant difference between both lures (paired t-test, p > 0.05, N = 8). Both Petri dishes were covered with red lid with large holes on the side to provide access to the lures.

In the comparison of OPP with DrosaLure™, we observed a slightly higher fly capture in the OPP Petri dish (17 ± 5.7; N = 8, Fig. 6) compared to the Drosalure™ Petri dish (13 ± 4.7; N = 8). However, this difference in average fly count was not significant (paired t-test, p > 0.05, N = 8).

## Discussion

The effectiveness of OPP (produced from *Citrus sinensis*) primarily relies on two key properties: attractiveness and toxicity. Both of these properties are evidently high for *D. suzukii*, explaining the high number of deceased fly individuals inside the Petri dishes containing OPP, in contrast to the small number of deceased flies in the hydrogel Petri dishes (Fig. 1 and 2A). Furthermore, dilution of OPP with tap water resulted in a strong reduction in the number of deceased flies (Fig. 5A), which highlights the attractiveness of undiluted OPP to *D. suzukii*. Our finding aligns with studies demonstrating the high attractiveness of oranges and orange scent to fruit flies^51,56,57^. The inherent preference of fruit flies for oranges may potentially be linked to the need to escape parasitism from endoparasitoid wasps strongly repelled by the smell of citrus^58^. Thus, the repellent effect of oranges for parasitoid wasps may explain the strong attraction of fruit flies to the scent of oranges at an ultimate level.

The attractiveness of OPP to *D. suzukii* is likely induced by fruit esters (e.g., neryl acetate, octyl acetate) and other volatile substances such as β-citronellal, β-myrcene, β-caryophyllene, linalool, pinene, terpinene, and citral contained in orange peels^48,59^. Especially, β-myrcene and β-linalool are known to be attractive to *D. suzukii*^51,52,57^. In contrast, the cyclic monoterpene D-limonene was found to be a potent repellent agent against *D. suzukii*^60^, whereas L-limonene was found to be attractive^61^. Since D-limonene is present in high quantities in orange peel extracts^48^, other volatile substances seem to override the repelling effect of this monoterpene. On overall, our results suggest that OPP used in an attract-and-kill approach, likely reduces the number of eggs laid in wine grapes located in the near of fruit fly traps because we found that 6 days old OPP still attracts and kills fruit flies (Fig. 4A). More importantly, we found a significant reduction of larvae in berries located adjacent to the OPP Petri dish (Fig. 3) and a higher number of dead females in the OPP Petri dish (Fig. 4B). The latter result is surprising given the fact that the sex ratio was 50:50% at the start of the experiment with important consequences for fruit fly control.

Our findings unveil that the combination of certain substances present in orange peels, along with the high salt concentration, exerts lethal effects on *D. suzukii*. This is evident from the reduction in the number of deceased fly individuals after 4 days, as illustrated in Fig. 5A and Fig. 5B, following the dilution of OPP and reduction of the salt concentration. High concentrations of NaCl lead to aversive behavior in fruit flies (e.g.,^62^), but in combination with OPP flies do not avoid a lure containing 10% NaCl as demonstrated by our choice experiments. The salt dilution experiment also suggests a possible contribution of NaCl to the toxic effect exerted by OPP.

In a modification of our choice experiment, we found OPP to be similar attractive compared to Drosalure™ (Fig. 6), predominantly containing vinegar, yeast and red wine. Consequently, it is plausible that in the field OPP has comparable attractiveness to this commercially-available lure when provided in attract-and-kill traps. However, it will be necessary to restrict OPP production to organically grown oranges in order to keep chemical pesticides away from this natural biocide. In this context, red wine (especially Merlot) and vinegar were identified as attractive lure for *D. suzukii* yielding better results than ethanol or acidic acid^63^. Therefore, chemicals in vinegar in addition to acetic acid, and substances in wine in addition to ethanol, serve as attractant for *D. suzukii*. Similarly, concord grape juice was found to be more attractive to both male and female *D. suzukii* than commercially available attractants such as “AlphaScents® SWD lure” and “Scentry® SWD lure”^64,65^. Furthermore, diluted concord grape juice attracted more female *D. suzukii* in the field compared to the commercially available attractants “Suzukii Trap® Max Captures,” “Trécé broad spectrum PEEL-PAK® multi-component lure,” and “Trécé high selectivity 3-component lure”^65^. On the contrary, orange and banana was identified a better food-bait for *D. suzukii* compared to wine or vinegar in the field^57^.

In a next step, it will be necessary to standardize the extraction procedure of orange peels to achieve reproducible results. Also, future outdoor experiments will be crucial in demonstrating the efficacy of OPP and other lures in real-world settings, where factors like wind, rain and temperature exert significant influence on fruit fly control. In such field scenarios, enhancing the attractiveness of OPP by adding additional attractive substances may be necessary to deter this fruit fly species from certain target fruits.

In conclusion, our salty orange peel product (OPP) was found to be attractive and toxic for *Drosophila suzukii* and reduced the number of eggs laid by females in ripe red wine grapes. The comparison with a commercially-available lure (DrosalureTM) in a laboratory setup revealed high potential as an effective and sustainable biocide in an attract-and-kill approach for the control of *Drosophila suzukii* in viticulture. Although OPP is toxic for *D. suzukii*, it is safe for various bee species as long as small entry holes prevent their access to fly traps containing this biocide. OPP is an upcycled product that can be readily produced from industrial waste materials accumulating during the production of organic orange juice. Future studies are needed to assess the effectiveness of OPP in real-world conditions and to compare its unintended bycatch with that of other lures.

## Materials and methods

### Insect breed

*Drosophila suzukii* individuals used in this study were grown in small plastic bottles sealed with a soft foam (Carolina^®^ Biological Supply Company, Burlington, North Carolina, USA)*,* at the Institute of Zoology in Graz (Austria). Instant Drosophila Medium (Carolina^®^ Biological Supply Company, Burlington, North Carolina, USA) was mixed with tap water and dried yeast (Carolina Dried Yeast C19010) to feed the flies. The individuals were maintained with fresh food every 3 days. The light/dark cycle was 12:12 h, and the average room temperature was 25.5 ± 1 °C (mean ± SD). The relative humidity was between 45–50%.

### Preparation of the biocide extract from orange peels

Organically farmed oranges, cultivated in South Italy, were bought and their peels were removed for the production of a botanical biocide. First, peels were cut into small pieces and submersed for two hours in an aqueous solution containing 10 w/v% NaCl. Then, the excess of this solution was removed with the help of towels and orange snippets were shredded using an electric slicer. This kind of paste was put into a fine-mesh nylon to manually squeeze orange peels. Between 10 to 15 ml of this extract was poured on top of a Petri dish (with a diameter of 13 cm) containing 90 ml hydrogel, which was produced by adding 90 ml water to 1.05 g of Aqua-Water-Granulat Terrabest (Feeders & more GmbH, Germany). Hydrogel delays desiccation of OPP and favored the release of volatile substances that are attractive for fruit flies. The Petri dishes were used fresh or 3–6 days later in two-choice experiments. Meanwhile this orange peel product (OPP) was stored at room temperature. Adding NaCl to the aqueous solution containing orange peel pieces may have an effect on the count of dead flies. To study the effect of adding NaCl to submersed orange peel pieces, NaCl was added in concentrations of 1 w/v %, 5 w/v % and 10 w/v % in an additional experiment.

### Two choice tests and larvae count

We conducted choice experiments by presenting two red wine grapes (each consisting of 10 berries) to 40 *D. suzukii* individuals in a terrarium together with Petri dishes containing either OPP or hydrogel. In this experiment, one grape was placed above Petri dishes containing OPP while the other one was placed above hydrogel, which served as the control. Organically grown red wine grapes (Frutura Obst & Gemüse Kompetenzzentrum GmbH, Hartl b. Kaindorf, Austria) were used as fruit target in choice experiments after carefully washing the berries with tap water prior to each experiment. The two grape clusters were positioned at a distance of 75 cm next to each other. The distance of berries to Petri dishes located below was 60 cm centimeters and the distance between both Petri dishes was 70 cm. In this experiment, we counted the number of dead fly individuals found in both Petri dishes on a daily basis and the number of fly maggots inside the berries were counted under a microscope after 4 days. The Petri dishes in all choice experiments were covered with a lid of identical red color (diameter 22 cm, height 4 cm) with four large holes on the side (10 cm × 2 cm) to prevent flies from selecting between Petri dishes on the basis of color. We selected red color lids for Petri dishes because from lab and field experiments it is known that *D. suzukii* prefers traps with red or dark color^66,67^. We randomized the positions of OPP and hydrogel Petri dishes in the terrarium to prevent side-bias in our choice experiment. For this purpose, the position of OPP Petri dishes were selected according to a binary random number list that was obtained with the help of a Python program code. All choice experiments were performed in a glass terrarium that was exposed to a light/dark cycle of 14:10 h (Juwel Aquarium LED Natural 6500 K plus LED Day 9000 K 11-Watt). During choice experiments, the temperature was 25.5 ± 1 ℃ (mean ± SD) and the relative humidity 42 ± 11% (mean ± SD). At the end of choice experiment, berries were cut into pieces and submerged in 250 mL aqueous salt solution (10 w/v% NaCl) to facilitate larvae count under a stereo microscope. This salt water treatment caused the larvae to float on top of the water surface.

### Comparision with droslure™

In an additional choice experiment, the hydrogel on the control side was replaced by 90 ml liquid Drosalure™ to compare the attractiveness of this commercially-available lure with OPP in the presence of ripe grape berries. Liquid Drosalure™ was bought from Andermatt Biogarten Inc. (Switzerland, Grossdietwil) and mainly consists of red wine, vinegar and yeast. Both, OPP and undiluted Drosalure™ were presented to *D. suzukii* in a terrarium below ripe red wine grapes.

### Statistical tests

All statistical tests have been performed in R Studio (Version 23.12.1, Posit Software, PBC). Fly count in Petri dishes and the sex ratio of dead flies was tested for statistical difference by application of a paired t-test. The normality of data distribution was tested with a Shapiro–Wilk test.

## Data Availability

The datasets used and/or analysed during the current study available from the corresponding author on reasonable request.
