# Publisher Correction: A novel sustainable biocide against the fruit fly *Drosophila suzukii* made from orange peels

**DOI:** 10.1038/s41598-024-84235-0

**Published:** 2025-01-07

**Authors:** Giovanni Davide Barone, Manfred Hartbauer

**Affiliations:** Department of Biology, University of Graz, Universitätsplatz 2, 8010 Graz, Austria

Correction to: *Scientific Reports* 10.1038/s41598-024-75365-6, published online 14 November 2024

The acknowledgements section in the original version of this Article was omitted. The acknowledgements section now reads:

“Thanks to Dr. Konstantinos Kostarakos who paved the path towards sustainable fruit fly control based on orange peels.”

The original Article has been corrected.

